# Pennsylvania

**Published:** 1899-07

**Authors:** 


### PENNSYLVANIA.

The Keystone State organization will hold its semi-annual meeting in the Lehigh Valley—meeting at Scranton, one of the busiest commercial centres of this valley of industry, enterprise and wealth, where has been drawn a goodly number of our profession, and where there should be to-day one of the strongest local associations in Pennsylvania. Scranton will afford another opportunity for central Pennsylvania to add to its quota of membership, and thus increase its interest and power in veterinary association circles. It will there discuss the questions of state and local interest, and learn of the continued progress in veterinary sanitary work throughout the Commonwealth. Every member of the profession will be welcomed to its deliberations, and every veterinarian will profit much by commingling with his fellow-workers, and getting in closer touch with the profession's progress, that offers so many new lines of employment.

